# Nutritional Status and Associated Factors Among Adult Gastrointestinal Cancer Patients at a Tertiary Hospital of Ethiopia: A Cross‐Sectional Study

**DOI:** 10.1002/cnr2.70597

**Published:** 2026-06-11

**Authors:** Mabratu Takele, Bati Leta, Abebe Dukessa Dubiwak, Mulualem Tadesse, Selam Tesfaye, Belay Zawdie

**Affiliations:** ^1^ Department of Biomedical Sciences, Faculty of Medical Science, Institute of Health Jimma University Jimma Ethiopia; ^2^ School of Medical Laboratory Sciences, Faculty of Medical Science, Institute of Health Jimma University Jimma Ethiopia; ^3^ Department of Oncology, Faculty of Medical Science, Institute of Health Jimma University Jimma Ethiopia

**Keywords:** associated factors, Ethiopia, gastrointestinal cancer, nutritional status

## Abstract

**Background:**

Malnutrition is increasingly recognized as a key prognostic factor in cancer patients. Although existing studies have reported malnutrition across various cancer types in Ethiopia, the nutritional status of the patients with gastrointestinal (GI) cancers remains largely unexamined.

**Aim:**

Therefore, the aim of this study was to assess nutritional status and identify its associated factors among adult patients with GI cancers at a tertiary hospital in Southwest Ethiopia.

**Methods:**

We conducted a facility‐based cross‐sectional study on patients diagnosed with GI cancer at Jimma University Medical Center. Nutritional status was determined using the Patient‐Generated Subjective Global Assessment (PG‐SGA) tool. Multivariable logistic regression analysis was used to determine the association between nutritional status and potential risk factors. A *p*‐value less than 0.05 with a 95% confidence interval was used to determine statistical significance.

**Results:**

Our study revealed that 74.4% (95% CI: 67.3–80.7) of adult GI cancer patients were at risk of malnutrition. The multivariable analysis indicated that being female [AOR = 3.76; 95% CI: (1.63–8.67)], having stage IV cancer [AOR = 4.10; 95% CI: (1.16–14.32)], having an illness duration of 25 months or more [AOR = 3.68; 95% CI: (1.42–9.57)], having a poor performance status [AOR = 2.72; 95% CI: (1.01–7.34)], and having comorbidities [AOR = 3.16; 95% CI: (1.33–7.50)] were significantly associated with an increased risk of malnutrition.

**Conclusion:**

Prevalence of malnutrition was high among adult GI cancer patients. The findings emphasize the crucial necessity for healthcare professionals to undertake routine nutritional screening and early nutritional interventions to enhance patient outcomes. Future research using a multicenter longitudinal study is recommended to build on these findings.

**Abbreviations**
AOR: adjusted odds ratio
COR: crude odds ratio
GIT: gastrointestinal tract
JUMC: Jimma University Medical Center
PG‐SGA: Patient‐Generated Subjective Global Assessment

## 1 Introduction

Cancer, characterized by uncontrolled proliferation of abnormal cells and invasion of surrounding tissues, remains a major global health challenge [1]. GI cancers, which arise from digestive organs including the esophagus, stomach, liver, pancreas, colon, and rectum, constitute a particularly lethal group of cancers [2]. Globally, GI cancers account for approximately 26.6% of global cancer incidence and 36.6% of cancer‐related mortality [3, 4]. In recent decades, there has been a major rise in the incidence of GI cancers in low‐ and middle‐income countries. This growing trend is driven by the complex interplay of demographic transitions, increased risk factor exposure, and limited access to preventive services and early diagnostic facilities [5]. In Ethiopia, existing evidence suggests that GI cancers contribute substantially to cancer‐related morbidity and mortality, thereby imposing a considerable burden on the healthcare system [6].

Malnutrition is one of the most common and clinically significant complications among patients with cancer, profoundly impacting treatment tolerance, disease progression, and survival. Evidence indicates that poor nutritional status is associated with approximately a twofold increase in mortality risk and poorer overall prognosis among cancer patients [7, 8]. Malnutrition is also linked to reduced tolerance to anticancer therapies, increased treatment‐related toxicity, prolonged hospital stays, and diminished quality of life. Furthermore, compromised nutritional status can impair immune function and increase susceptibility to infections [9, 10, 11].

The prevalence of malnutrition is particularly high among patients with GI cancer. A recent systematic review and meta‐analysis reported that malnutrition affects approximately 61% of adult GI cancer patients, with prevalence reaching 78% in upper GI tract cancers [8]. The mechanisms underlying cancer‐related malnutrition are multifactorial. Tumor‐related factors such as mechanical obstructions and nutrient malabsorption contribute significantly to reduced nutritional intake and utilization [12, 13]. Additionally, GI cancer patients often experience gastrointestinal symptoms and treatment‐related side effects, which further compromise dietary intake and nutritional status [14, 15].

The burden of malnutrition among cancer patients is particularly severe in low‐ and middle‐income countries. In many Sub‐Saharan African countries, including Ethiopia, cancer is frequently diagnosed at advanced stages of the disease. Late‐stage diagnosis often leads to deterioration in nutritional status, while widespread food insecurity and limited healthcare infrastructure further exacerbate the risk among oncology patients [16, 17]. Previous studies conducted in Ethiopia have reported malnutrition prevalence ranging from 17% to 86% among cancer patients [18]. Despite evidence on the nutritional status of patients with various types of cancer, data specific to those with GI cancers in Ethiopia remain limited. This gap hinders efforts to improve the management of cancer‐related malnutrition and patient outcomes in resource‐limited settings.

Therefore, this study aimed to assess the nutritional status of adult patients with GI cancer and to identify factors associated with malnutrition at Jimma University Medical Center (JUMC), the sole tertiary referral hospital serving Southwest Ethiopia. Nutritional status was evaluated using the PG‐SGA, which demonstrates superior sensitivity in identifying malnutrition in oncology populations [19]. This study contributes to the existing body of knowledge on malnutrition prevalence and its determinants in GI cancer patients. Additionally, the findings may assist healthcare professionals in prioritizing strategies for malnutrition prevention and early detection among cancer patients. Overall, the study provides evidence that may reduce the GI cancer‐related morbidity and mortality in a clinical setting.

## 2 Methods

### 2.1 Study Design, Area, and Period

A facility‐based cross‐sectional study was conducted at the oncology department of JUMC from April 1, 2024 to June 30, 2024. JUMC is the only teaching and referral hospital found in Oromia regional state, Southwest Ethiopia. It is found at a distance of 350 km from Addis Ababa, the capital city of Ethiopia. The hospital provides health services to the catchment population of about 15 million people. JUMC's oncology unit is the only tertiary public facility in southwest Ethiopia that provides cancer diagnosis and treatment.

### 2.2 Population and Eligibility Criteria

The study population consisted of all adult patients diagnosed with GI cancers who attended the oncology department of JUMC during the data collection period. Individuals aged 18 years and above who were capable of providing written informed consent and were receiving cancer treatment were eligible for inclusion. Patients who had severe physical or mental conditions that prevented their participation were excluded from the study.

### 2.3 Sample Size Determination and Sampling Technique

The required sample size was initially estimated using the single population proportion formula with the following assumptions: a 95% confidence level (Z_1₋α_/2 = 1.96), a margin of error of 5%, and a previously reported prevalence of malnutrition among GI cancer patients of 77.6% [20]. Based on these parameters, the calculated sample size was 267 participants. However, because the total number of eligible patients available during the study period was 176, all eligible individuals were included in the study.

### 2.4 Study Variables

#### 2.4.1 Dependent Variable

- The nutritional status of adult GI cancer patients.

#### 2.4.2 Independent Variables

- *Socio‐demographic characteristics:* Age, sex, level of education, residence, marital status, and occupation.
- *Clinical characteristics:* Type of cancer, stage of cancer, duration of illness, cycle of chemotherapy, treatment adherence, comorbidity, and performance status.
- *Life style factors:* Consumption of alcohol, cigarette smoking, and khat chewing.

### 2.5 Operational Definitions

- *Treatment adherence:* Treatment adherence was defined by combining patient's self‐report and clinical records. Good treatment adherence was defined as taking at least 80% of prescribed chemotherapy cycles received on schedule, while poor treatment adherence was defined as taking less than 80% of their medication [21].
- *Comorbidity:* Comorbidity is defined as the presence of one or more documented chronic medical conditions co‐existing alongside the primary index illness.
- *Duration of illness:* Duration of illness is defined as the total elapsed time from the date of the patient's first clinical diagnosis to the date of enrollment in this study.

### 2.6 Data Collection Process and Data Quality Control

The socio‐demographic, life style, and clinical variables were collected using face‐to‐face interviews and review of patients' medical records. Nutritional status was evaluated using the PG‐SGA tool. Therefore, patients with a total score of less than 9 were categorized as well‐nourished, while those with scores of 9 or more were classified as at risk of malnutrition [22, 23]. The performance status was assessed using the Eastern Cooperative Oncology Group performance scale [24]. A cut‐off point of 2 was applied, where scores less than 2 indicated good performance status, while scores of 2 or above were considered poor performance status.

To maintain data quality, a 2‐day training session was provided to data collectors (two BSc nurses) and one supervisor (an MSc holder in human nutrition). The training focused on the study objectives, ethical considerations, and standardized data collection procedures. Throughout the data collection period, continuous supervision and close monitoring were carried out by the principal investigator and the designated supervisor.

### 2.7 Data Processing and Statistical Analysis

Data were first entered into Epi Data version 4.2 and subsequently exported to SPSS version 26.0 for analysis. Continuous variables were summarized using means and standard deviations, while categorical variables were described using frequencies and percentages. The results were summarized and presented by statements, tables, and figures.

Bivariable logistic regression was performed to compute crude odds ratio (COR) and to identify candidate variables for multivariable analysis at a significance level of *p* < 0.25. Variables meeting this criterion were entered into a multivariable logistic regression model to determine independent predictors of nutritional status. Statistical significance was declared at *p* < 0.05 with a 95% confidence interval, and the magnitude of associations was expressed using adjusted odds ratio (AOR). Multicollinearity among independent variables was assessed using the variance inflation factor (VIF). The VIF values ranged from 1.04 to 1.62, indicating the absence of multicollinearity among the independent variables. Model adequacy was evaluated using the Hosmer–Lemeshow goodness‐of‐fit test, where a *p*‐value greater than 0.05 suggested a good model.

## 3 Results

### 3.1 Socio‐Demographic Characteristics of the Study Participants

A total of 176 adult patients diagnosed with GI cancers participated in the study. Of these, 99 (56.2%) were male. The mean age of the participants was 48 years (standard deviation of 9.5). Regarding residence, 89 (50.6%) of the participants were from rural areas, which was nearly comparable to those residing in urban settings. Among the total respondents, 124 (70.5%) were married and 75 (42.6%) were farmers. With respect to educational level, 76 (43.2%) had not attended formal education and only 25 (14.2%) had completed higher education (Table 1).

**TABLE 1:** Socio‐demographic characteristics of adult GI cancer patients at JUMC, Jimma, Southwest Ethiopia, 2024.

Variables	Frequency (*n* = 176)	Percent
Age (years)		
< 40	43	24.4
40–60	78	44.3
≥ 60	55	31.3
Sex		
Male	99	56.2
Female	77	43.8
Place of residence		
Rural	89	50.6
Urban	87	49.4
Marital status		
Single	52	29.5
Married	124	70.5
Level of education		
No formal education	76	43.2
Primary	48	27.3
Secondary	27	15.3
Collage/University	25	14.2
Occupation		
Farmer	75	42.6
Merchant	29	16.5
Government employee	25	14.2
Self‐employee	47	26.7

### 3.2 Clinical and Life Style Factors of the Study Participants

In this study, colorectal cancer was the most prevalent type of gastrointestinal malignancy, representing nearly one‐third (32.4%) of the cases. This was followed by gastric cancer, which accounted for 23.9% of the patients. Among the study participants, most of the patients were diagnosed at Stage III 60 (34.1%), followed by Stage IV 55 (31.3%). With respect to illness duration since diagnosis, 80 (45.5%) patients had the disease for less than 12 months. More than half of patients 94 (53.4%) had undergone their first cycle of chemotherapy. However, only 59 (33.5%) had good treatment adherence.

In terms of comorbidity, 63 (35.8%) of the patients had comorbid illnesses. Among the respondents, a higher proportion of them had hypertension 24 (13.6%). Furthermore, the majority of the study participants had poor performance status 111 (63.1%). Of the total respondents, 58 (33.4%) had a history of khat chewing, 51 (29.0%) had a history of alcohol intake, and 15 (8.5%) had a history of cigarette smoking (Table 2).

**TABLE 2:** Clinical and life style factors of adult GI cancer patients at JUMC, Jimma, Southwest Ethiopia, 2024.

Variables	Frequency (*n* = 176)	Percent
Cancer type		
Colorectal Cancer	57	32.4
Gastric Cancer	42	23.9
Liver Cancer	34	19.3
Esophageal Cancer	26	14.8
Pancreatic Cancer	17	9.6
Stage of cancer		
Stage I	27	15.3
Stage II	34	19.3
Stage III	60	34.1
Stage IV	55	31.3
Duration of illness		
< 12 months	80	45.5
12–24 months	41	23.3
≥ 25 months	55	31.2
Cycle of chemotherapy		
First	94	53.4
Second	24	13.6
Third	26	14.8
Fourth and above	32	18.2
Treatment adherence		
Good	59	33.5
Poor	117	66.5
Comorbidity		
No	113	64.2
Hypertension	24	13.6
Chronic kidney disease	15	8.5
Diabetes mellitus	13	7.4
Chronic liver diseases	11	6.3
Performance status		
Good	65	36.9
Poor	111	63.1
Alcohol use		
Yes	51	29.0
No	125	71.0
Cigarette smoking		
Yes	15	8.5
No	161	91.5
Khat chewing		
Yes	58	33.0
No	118	67.0

### 3.3 Nutritional Status of Adult GI Cancer Patients

**FIGURE 1:**
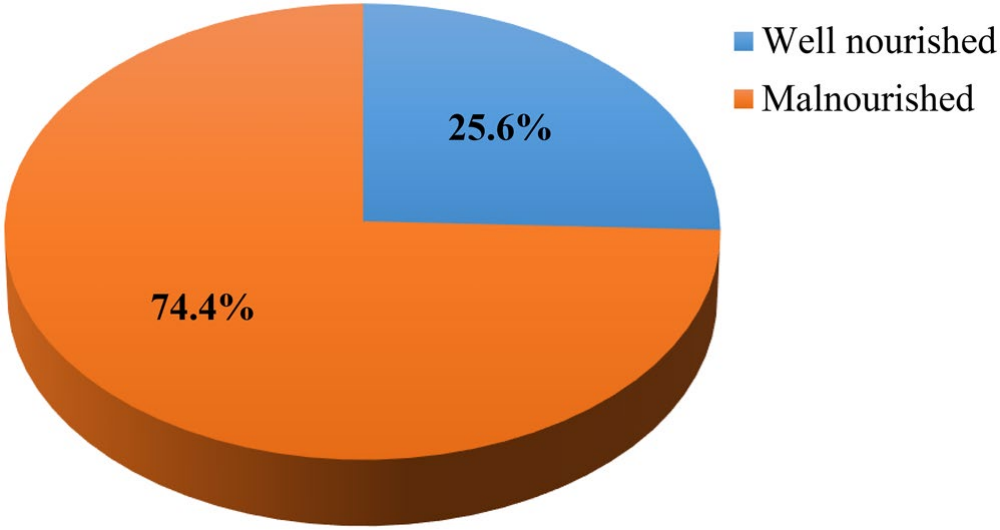
Nutritional status of adult GI cancer patients at JUMC, Jimma, Southwest Ethiopia, 2024.

The findings of this study indicated that 131 (95% CI: 67.3–80.7) of study participants were at risk of malnutrition, while 45 (25.6%) were well nourished (Figure 1).

### 3.4 Factors Associated With Nutritional Status Among Adult GI Cancer Patients

In this study, multiple factors were associated with nutritional status in adult GI cancer patients. The bivariable logistic regression analysis showed that age, sex, place of residence, stage of cancer, performance status, comorbidity, duration of illness, alcohol use, and treatment adherence were significantly associated with nutritional status at *p* < 0.25 and entered into the multivariable logistic regression for further analysis. After adjusting for other variables, being female, Stage IV cancer, performance status, comorbidity, and duration of illness had a statistically significant association with risk of malnutrition in adult GI cancer patients.

Keeping other variables constant, being female was 3.76 times higher odds of being at risk of malnutrition compared with male patients [AOR = 3.76; 95% CI: (1.63–8.67)]. Similarly, patients diagnosed with Stage IV cancer were 4.10 times more likely to be at risk of malnutrition than those with Stage I disease [AOR = 4.10; 95% CI: (1.16–14.32)].

Furthermore, patients with a disease duration of 25 months or longer had 3.68 times greater odds of malnutrition compared with those whose illness duration was less than 12 months [AOR = 3.68; 95% CI: (1.42–9.57)]. Adult GI cancer patients who did have a poor performance status had 2.72 times higher odds of being malnourished compared to their counterparts [AOR = 2.72; 95% CI: (1.01–7.34)].

Finally, the presence of comorbid conditions significantly increased the risk of malnutrition. Patients with comorbid illnesses were 3.16 times more likely to experience malnutrition than those without comorbidities [AOR = 3.16; 95% CI: (1.33–7.50)] (Table 3).

**TABLE 3:** Results for the final logistic regression model of adult GI cancer patients at JUMC, Jimma, Southwest Ethiopia, 2024.

Variables		Nutritional status		COR (95% CI)	AOR (95% CI)
		Well nourished	Malnourished		
Age	< 40	16	27	1	1
	40–60	18	60	1.98 (0.88–4.45)	1.14 (0.43–2.97)
	≥ 60	11	44	2.37 (0.96–5.86)	1.85 (0.63–5.45)
Sex	Male	33	66	1	1
	Female	12	65	2.71 (1.29–5.70)	3.76 (1.63–8.67)^a^
Place of residence	Rural	17	72	2.01 (1.00–4.02)	1.84 (0.84–4.02)
	Urban	28	59	1	1
Stage of cancer	Stage I	11	16	1	1
	Stage II	11	23	1.23 (0.39–2.49)	1.84 (0.26–2.75)
	Stage III	13	47	2.49 (1.51–3.47)	2.18 (0.73–6.47)
	Stage IV	10	45	3.09 (2.06–4.12)	4.10 (1.16–14.32)^a^
Duration of illness	< 12 months	27	53	1	1
	12–24 months	9	32	1.81 (0.76–4.34)	2.34 (0.86–6.42)
	≥ 25 months	9	46	2.60 (1.11–6.10)	3.68 (1.42–9.57)^a^
Performance status	Good	22	43	1	1
	Poor	21	90	2.19 (1.29–2.89)	2.72 (1.01–7.34)^a^
Comorbidity	Yes	11	52	2.04 (0.95–4.37)	3.16 (1.33–7.50)^a^
	No	34	79	1	1
Treatment adherence	Good	24	35	1	1
	Poor	21	96	3.14 (1.55–6.33)	2.32 (0.82–6.55)
Alcohol use	Yes	8	43	2.26 (0.97–5.27)	1.76 (0.56–5.54)
	No	37	88	1	1

^a^ Indicates variables that were statistically significant at a *p*‐value of less than 0.05.

## 4 Discussion

Our study sought to assess the nutritional status and its associated factors among adult patients with GI cancer. We found that 74.4% of adult patients with GI cancer were at risk of malnutrition. This finding is comparable to findings reported in studies from Brazil (77%) [25] and Vietnam (77.6%) [20]. The results suggest that patients with GI cancer are highly vulnerable to malnutrition and require timely nutritional education and professional support. However, our finding is notably higher than those reported in studies from China (43.5%) [26] and Addis Ababa (58.2%) [27]. The high level of malnutrition in our study may be explained by differences in nutritional assessment tools, study populations, and the number of chemotherapy cycles. On the other hand, our finding is lower than that of a study conducted in China (98%) [28]. These discrepancies may be due to the fact that the latter study included GI cancer patients with advanced‐stage disease and poor performance status.

We also investigated factors that were significantly associated with a high risk of malnutrition in adult patients with GI cancers. Female patients had a 3.76‐fold higher likelihood of experiencing malnutrition risk than male patients. This finding is consistent with studies from Jordan [29] and Bangladesh [30]. The observed variations in nutritional status between sexes may be linked to distinct biological and sociocultural factors [31]. However, our finding differs from those reported in Malaysia [32] and China [28], where males had a higher risk of malnutrition. These inconsistent results highlight the need for further research to clarify whether sex‐specific metabolic responses influence malnutrition risk among cancer patients.

Cancer stage also showed a strong association with malnutrition risk. Patients with Stage IV cancer were approximately 4.1 times more likely to experience malnutrition than those with stage I disease. This finding is consistent with previous studies conducted in Ethiopia [27] and Bangladesh [33]. Disease progression and proliferating cancer cells associate with hypermetabolism, which correlates with depleted body nutritional reserves. Additionally, advanced tumors are more likely to associate with mechanical obstruction or severe GI symptoms, which can impair food intake and nutrient absorption [34].

The duration of illness was another significant factor influencing malnutrition risk. Patients who had lived with cancer for 25 months or longer were 3.68 times more likely to be at risk of malnutrition than those with disease duration of less than 12 months. Our finding is in line with research from Bangladesh [30]. Prolonged cancer treatment and metabolic alterations associate with tissue catabolism for energy during long‐standing illness, which correlates with a declining nutritional status [35].

Performance status was a significant predictor of malnutrition risk in patients with GI cancers. Individuals with poor performance status were 2.72 times more likely to have malnutrition compared to those with good performance status. Similar associations have been documented in studies from Ethiopia [36], Iran [37], and France [38]. This can be explained by the link between extended cancer therapies and systemic metabolic shifts with accelerated tissue breakdown for energy during chronic illness, which ultimately aligns with worsening nutritional depletion [39].

Furthermore, the presence of comorbid conditions significantly increased the likelihood of malnutrition. Patients with additional comorbidities had a 3.16‐fold higher risk of developing malnutrition than those without comorbidities. This finding is consistent with research from China [26]. This can be explained by the link between the high physical and economic demands of managing multimorbidity and the disruption of cancer treatments, which ultimately correlates with a high prevalence of malnutrition.

## 5 Strength and Limitations

To the best of our knowledge the present study is the first to evaluate the nutritional status of GI cancer patients using the PG‐SGA in Ethiopia. Despite its strengths, this study has also limitations that should be acknowledged. The single‐center design and relatively small sample size may limit the generalizability of the findings to broader populations. Moreover, the use of interviewer‐administered questionnaires may have introduced social desirability bias, as participants might have provided responses perceived as more socially acceptable rather than strictly accurate.

## 6 Conclusion

The results of this study indicate that adult patients with GI cancers have a high prevalence of malnutrition. We observed that female patients, Stage IV cancer, poor performance status, prolonged illness duration, and presence of comorbidities are risk factors for malnutrition in adult patients with GI cancer. To improve nutritional status of adult GI cancer patients, healthcare professionals need to provide nutritional education and professional support for patients who may be at risk of malnutrition. Healthcare facilities should implement routine nutritional screening and integrate early nutritional interventions to the malnourished patients. Further prospective studies consisting of larger cohorts and multicenter studies are needed to determine malnutrition and associated factors in adult patients with GI cancer.

## Author Contributions

**Bati Leta:** conceptualization, methodology, writing – review and editing, writing – original draft, validation, visualization, data curation. **Belay Zawdie:** conceptualization, methodology, writing – review and editing, validation, data curation, supervision, formal analysis. **Mabratu Takele:** conceptualization, methodology, writing – review and editing, writing – original draft, software, investigation, formal analysis, data curation. **Mulualem Tadesse:** conceptualization, methodology, writing – review and editing, project administration, resources. **Selam Tesfaye:** conceptualization, methodology, writing – review and editing, supervision. **Abebe Dukessa Dubiwak:** conceptualization, methodology, writing – review and editing, investigation, visualization.

## Funding

The authors have nothing to report.

## Ethics Statement

This study was approved by the Institutional Review Board at Jimma University (Reference number: JUIH/IRB/725/23, Date: December 29, 2023) and was conducted in accordance with the principle of the Declaration of Helsinki.

## Consent

Informed written consent was obtained from all individual participants included in the study.

## Conflicts of Interest

The authors declare no conflicts of interest.

## Data Availability

The data that support the findings of this study are available on request from the corresponding author. The data are not publicly available due to privacy or ethical restrictions.
